# A Rationale for Micro-textured Breast Implant Augmentation

**DOI:** 10.1093/asjof/ojac020

**Published:** 2022-03-30

**Authors:** Julia A Chiemi, S Sean Kelishadi

## Abstract

**Background:**

Textured breast implants have been used in aesthetic breast surgery to decrease rates of malposition and capsular contracture. Recent concerns regarding breast implant-associated anaplastic large cell lymphoma (BIA-ALCL)’s link to textured devices have prompted many physicians to reevaluate their use.

**Objectives:**

The authors aimed to create an algorithm for when to use smooth vs micro-textured breast implants and provide their rationale for when micro-textured implants may be more beneficial.

**Methods:**

In total, 133 patients received primary augmentations performed by a single surgeon from January 2018 to December 2020; 84 patients received smooth implants and 49 patients received micro-textured implants. All surgeries were performed in the dual plane using an inframammary incision. Implant-related complications and scar malposition were recorded and compared between groups.

**Results:**

No significant difference in the prevalence of implant-related complications was found (3.57% for smooth devices and 2.04% for micro-textured devices [*P*-value 0.621257; 95% CI −0.06100 to 0.007467]). There were no cases of BIA-ALCL. A comparison of scar malposition rates between the smooth and micro-textured groups also revealed no statistically significant difference (15.4% for smooth devices and 8.16% for micro-textured devices [*P*-value 0.226156; 95% CI −0.1200 to 0.007467]). Patients in the micro-textured group proportionately had more anatomical risk factors for malposition.

**Conclusions:**

Micro-textured breast implants continue to be a safe and effective choice for patients. Micro-textured implants show a trend toward decreased scar malposition, although not statistically significant. Patients at high risk for malposition with micro-textured breast implants give similar results to patients at average risk for malposition with smooth implants.

**Level of Evidence: 3:**

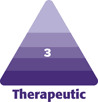

The advantages of breast implants with textured shells include decreased malposition, lower rates of capsular contracture, and lower rates of reoperation.^1,2^ The characteristics of surface texturization are classified as macro-texture, micro-texture, meso-texture, and nano-texture/smooth categories; each class of texture performs differently clinically, resulting in unique patterns of tissue ingrowth and encapsulation.^3-5^

More recently, concerns regarding breast implant-associated anaplastic large cell lymphoma (BIA-ALCL) have led to questions about the safety of textured implants and their place in modern plastic surgery. The scrutiny led to the FDA’s 2019 request to Allergan (Irvine, CA) to voluntarily recall their line of Biocell (Allergan, Irvine, CA) macro-textured breast implants in the United States; locally, many surgeons have discontinued their use of textured implants, and globally there are discussions about the safety of textured breast implants.^6^

Modern plastic surgery no longer relies on a cookie-cutter approach to breast augmentation. Plastic surgeons now have a multitude of implant options available to them to customize results while considering each patient’s breast anatomy, desired aesthetic results, and available options. Given the pros and cons of available implants, the authors have compiled their rationale for when the use of micro-textured silicone gel breast implants may be more beneficial.

## METHODS

A cohort analysis was conducted using data collected from 133 primary augmentation mammaplasty cases performed between January 2018 and December 2020. All surgeries were performed by the senior author (S.S.K.) and were consecutive patients. A total of 133 surgeries were performed with bilateral silicone gel breast implants, of which 84 cases utilized smooth breast implants and 49 cases utilized micro-textured breast implants. All patients were female, ranging from ages 18 to 64 years (mean patients age in this study was 32 years). 

**Figure 1.**
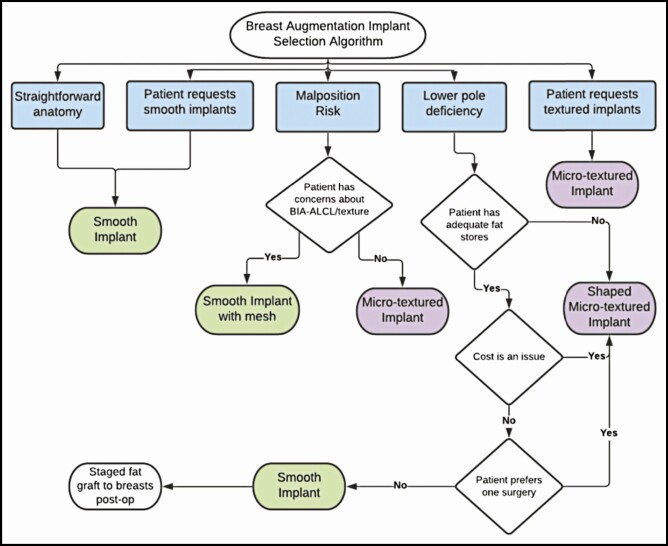
Clinical considerations taken into account when selecting implant surface and shape for patients.

Implant surface (micro-textured or smooth) and shape (round or anatomical/shaped) were selected for each patient at a consultation or preoperative appointment according to SSK’s Breast Augmentation Implant Selection Algorithm (Figure 1) based on individualized anatomy, patient requests and concerns, and aesthetic goals. Written consent was provided, by which the patients agreed to the use and analysis of their data.

In all breast implant cases, techniques to reduce the risk of bacterial contamination of the breast implants during surgery were employed.^7^ Intravenous antibiotics were administered to patients at the start of the anesthetic (2 g IV Cefazolin except where patients indicated an antibiotic allergy). All patients in the study received an inframammary incision for dual-plane breast augmentation. Careful atraumatic dissection with electrocautery was performed to have a bloodless field. Pocket irrigation was performed in all cases, with the preferred irrigation being a triple antibiotic solution containing Cefazolin, Bacitracin, and Gentamicin,^8^ except when patients had an allergy or if supplies of ingredients for the triple antibiotic were unavailable, in those situations.

PhaseOne (Nashville, TN) hypochlorous acid (HOCl) or 50% betadine solution was used. The use of an introduction sleeve, new gloves before handling the implants, and careful attention to sterile technique were all employed to minimize the bacterial burden. Three-layered suture closure was used for all cases. Drains were never used. Postoperative antibiotic prophylaxis was employed, with all patients receiving a cephalosporin antibiotic (Cephalexin 500mg, PO TID) for 10 days except in the case of a known allergy.

Patients were monitored from the time of implant placement with the typical in-person follow-up schedule of 1 week post-surgery, 1 month post-surgery, 3 months post-surgery, 6 months post-surgery, and yearly follow-up appointments for each subsequent anniversary. Patient photographs were reviewed at minimum 2 months follow-up and beyond, and an assessment was made of the patient’s outcomes from their latest available follow-up at the time of the study. Complications related to breast implants including skin infection, wound dehiscence, hematoma, seroma, capsular contracture (Baker Grade III-IV), and need for reoperation were recorded. Implant malposition was also gauged by comparing inframammary scar migration in postoperative appointment photographs.

**Figure 2.**
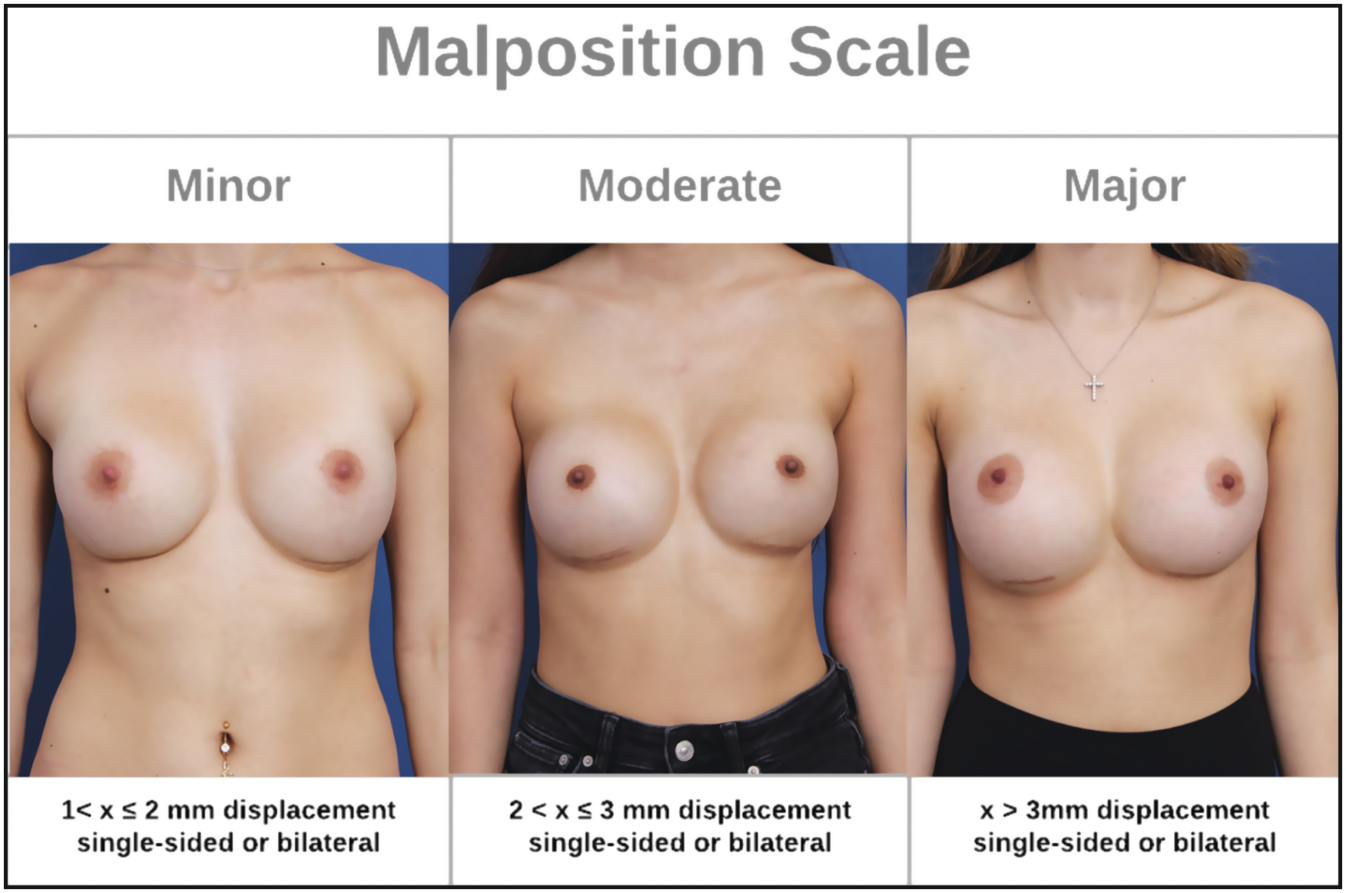
Study malposition classification scale based on scar movement from original inframammary fold placement in millimeters. Pictured (left to right): a 31-year-old female, a 24-year-old female, and a 28-year-old female.

The average length of follow-up for each group’s scar assessment was comparable to left-skewed distributions: 7.4 months for the smooth group and 8.2 months for the micro-textured group. For both groups, the mean follow-up was less than a year, and the maximum follow-up was 2 years. Measurements were taken on a computer by a single independent evaluator from the scar position to the visible inframammary crease in photographs. This was used as a gauge of displacement because all incisions were made directly in the inframammary crease during surgery and were secured there upon closure. A study scale was developed to classify scar malposition into 3 categories (Figure 2): minor (1 < x ≤ 2 mm, single-side or bilateral), moderate (2 < x ≤ 3 mm, single-side or bilateral), and major (x > 3 mm, single-side or bilateral). This scale was developed by the authors for this study and has not been utilized elsewhere. Statistical analyses to compare the complication and malposition rates of the smooth and textured implant groups were performed on a computer in Excel using unpaired 2-tailed *t*-tests, and 95% CIs were constructed.

## RESULTS

Following the Breast Augmentation Implant Selection Algorithm (Figure 1), 63.2% of patients (84) were placed into the smooth implant group, while 36.8% (49) were placed into the micro-textured implant group (Table 1). Of the smooth implant group, 52 patients received implants from Allergan’s Natrelle Inspira SoftTouch Smooth Round Gel line, while 31 received Sientra HSC + Opus Luxe Smooth implants (Santa Barbara, CA) and 1 patient received Mentor MemoryGel Smooth Round implants (Irvine, CA). All patients in the micro-textured implant group (with the exception of 1 patient who received Mentor’s Siltex MemoryShape implants due to a sizing preference) received implants from Sientra’s Opus Luxe line; 28 patients received round implants, while 20 patients received classic base shaped implants (Table 2). The average implant size between groups was 435 ccs for the smooth group and 411 ccs for the micro-textured group. 

**Table 1.** Proportions of Implant Surface Type Used and Total Group Numbers

Implant type	Primary augmentation-mammaplasty (n =133)
Smooth implants	63.2% (84)
Micro-textured implants	36.8% (49)

**Table 2.** Proportions and Total Counts of the Different Implant Brands and Styles Received by Patients in the Cohort

Implant type	Primary augmentation-mammaplasty (N = 133)	Percentage
Allergan Smooth Round (Irvine, CA)	52	39.1%
Sientra Smooth Round (Santa Barbara, CA)	31	23.3%
Sientra Round Micro-textured (Santa Barbara, CA)	28	21.1%
Sientra Shaped Micro-textured (Santa Barbara, CA)	20	15.0%
Mentor Smooth Round (Irvine, CA)	1	0.8%
Mentor Shaped Micro-textured (Irvine, CA)	1	0.8%

The mean follow-up time period was 9.3 months (SD 1.23) for the smooth devices alone cohort, and 8.6 months (SD 1.04) for the micro-textured devices cohort. No significant difference in the prevalence of implant-related complications was found between the smooth and micro-textured groups (*P-*value 0.621257; 95% CI −0.06100 to 0.007467). The scaled complication rate for the smooth group was 3.57% (3) compared with 2.04% (1) for the micro-textured group (Table 3). Though not considered a formal complication in this study, the senior author wished to evaluate the prevalence of clinically significant scar malposition between the 2 study groups to test the claim that textured implants are effective at preventing malposition. No statistically significant difference was found in the prevalence of scar malposition between the smooth and textured groups, with 15.4% of patients with smooth implants developing scar malposition compared with 8.16% of patients with textured implants (Table 4) (*P*-value 0.226156; 95% CI −0.1200 to 0.007467). The micro-textured cohort demonstrated a reduced severity of scar malposition when it did occur, with a higher proportion of its malposition cases classified as minor compared with the smooth implant group. The textured implant group had no cases of major scar malposition and only one case of moderate scar malposition.

**Table 3.** Total Incidence and Proportions of Implant-Related Complications Recorded Between the Smooth and Micro-Textured Implant Groups Throughout the Duration of Follow-Up

Complication type	Smooth implants (n = 84)	Micro-textured implants (n = 49)
Hematoma	2	0
Seroma	0	0
Implant extrusion	1	1
Wound dehiscence	0	0
Skin infection	0	0
Capsular contracture	0	0
Need for reoperation, other	0	0
Total (no. of complications and % of cohort w/complications)	3 (3.57%)	1 (2.04%)

**Table 4.** Incidence, Scaled Prevalence Rates, and Total Prevalence of Implant Malposition Recorded Between the Smooth and Micro-Textured Implant Groups Throughout the Duration of Follow-Up

Implant type	Minor malposition (1 < x ≤ 2 mm)	Moderate malposition (2 < x ≤ 3 mm)	Major malposition (>3 mm)	Total	Prevalence by cohort
Smooth implants (n = 84)	2	7	4	13	15.4%
Allergan (n = 52) (Irvine, CA)	0	6	3	9	17.3%
Sientra (n = 31) (Santa Barbara, CA)	2	1	1	4	12.9%
Micro-textured implants (n = 49)	3	1	0	4	8.16%
Total	5	8	4	17	12.7%

## Discussion

The results from this analysis of a single-surgeon cohort study indicate that textured breast implants continue to be a safe and effective choice for patients. No statistically significant difference was found in implant-related complication rates between the smooth and textured implant groups. These findings are consistent with existing safety data on micro-textured breast implants and their longitudinal clinical performance.^9-12^

This study found no significant difference in the prevalence of clinically significant malposition between the smooth and micro-textured implant groups. This was contrary to the expected result of a decreased rate of malposition for the textured subset; it is possible with a larger sample size that a statistical benefit of micro-textured implants could have been seen. Additionally, this study utilized only micro-textured implants as these are the only textured devices available in the US market. Due to data showing higher incidence of double capsule formation and BIA-ALCL linked to Allergan’s macro-textured Biocell implants,^13-15^ the senior author chose to never implant these devices in patients, and all textured devices used in this cohort were from Sientra’s Opus Luxe or Mentor’s Siltex micro-textured lines. More aggressively textured devices have been shown to provide high stability and immobility within the breast pocket due to a greater coefficient of friction. However, macro-texture facilitates deeper ingrowth of the surrounding tissue (the “Velcro effect”) and has been linked to higher rates of double capsule formation, late seroma, and BIA-ALCL. A significant reduction in scar malposition rates may have been observed if implants with a higher grade of texture had been used, but the authors chose to use only micro-textured implants due to safety considerations given the existing data on these shells.

**Figure 3.**
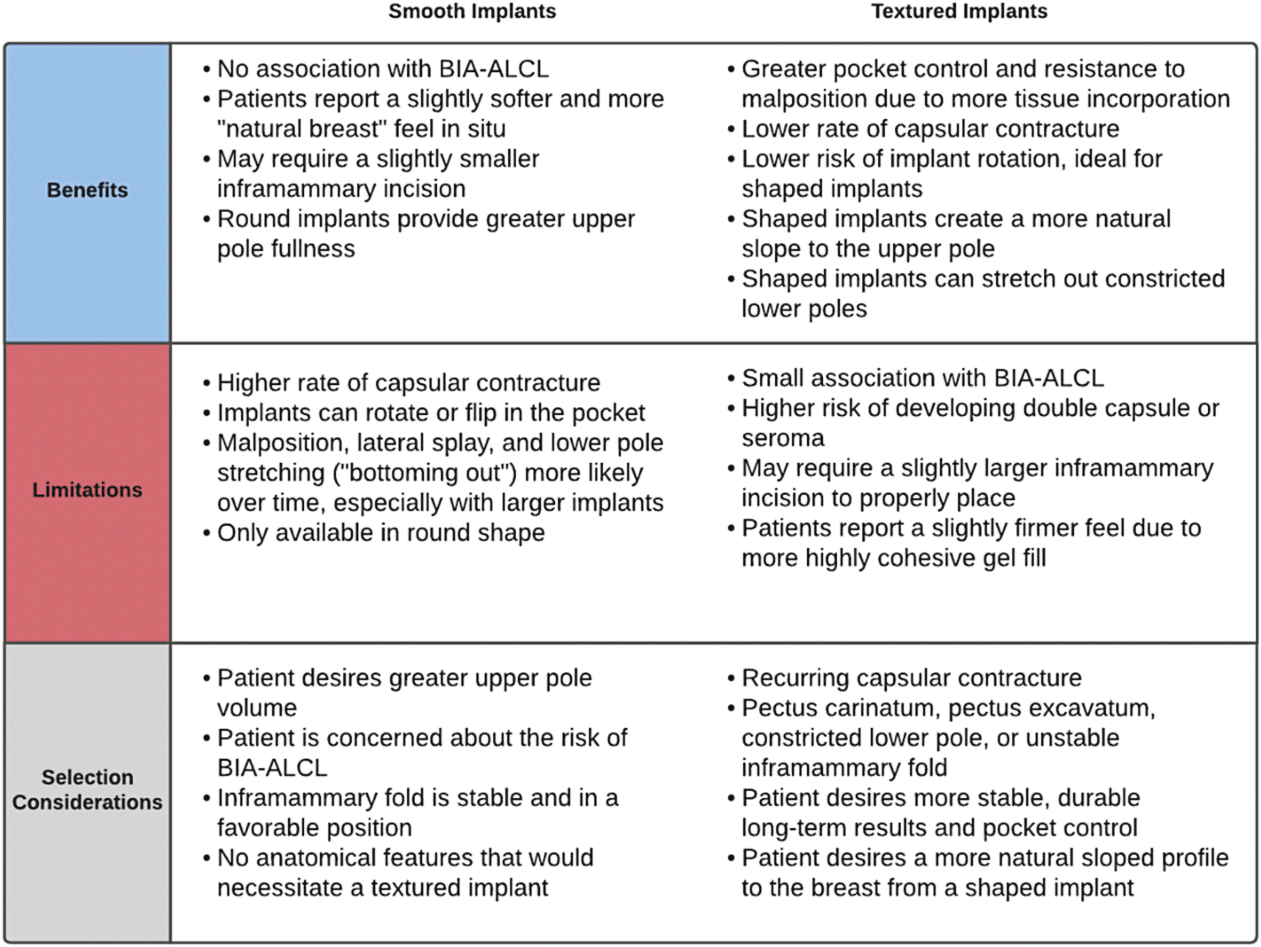
A summary of the benefits, limitations, and selection considerations for smooth and textured silicone implants. BIA-ALCL, breast implant-associated anaplastic large cell lymphoma.

One of the reported benefits of textured breast implants is their adherence to the pocket provided by the implant surface’s higher surface area and coefficient of friction.^1,9,16^ This resistance to movement is the main implant selection consideration when choosing between smooth and micro-textured implants for individual patients^11,17-19^ (Figure 3). However, despite the lack of a statistically significant difference in the malposition rates of this study cohort, the authors believe that the micro-textured implants were still effective as a protective measure and served to fully prevent major malposition. Further analysis of the data revealed that a higher proportion of patients in the micro-textured group possessed anatomical risk factors for malposition before surgery. These risk factors included features of pectus carinatum, pectus excavatum, or an unstable inframammary fold. While micro-textured implants were ultimately not fully protective against developing malposition and unfavorable scar position in these patients, the authors believe that the malposition observed in these patients could have been more severe had they received smooth implants instead. The authors note that these judgments of the patients in the micro-textured cohort being, on average, more challenging cases are merely their clinical opinion, and these differences were not stratified through the methodology. In the end, patients possessing anatomical factors that placed them at higher risk for developing malposition gave similar results with micro-textured implants to patients at average risk for malposition with smooth implants.

**Figure 4.**
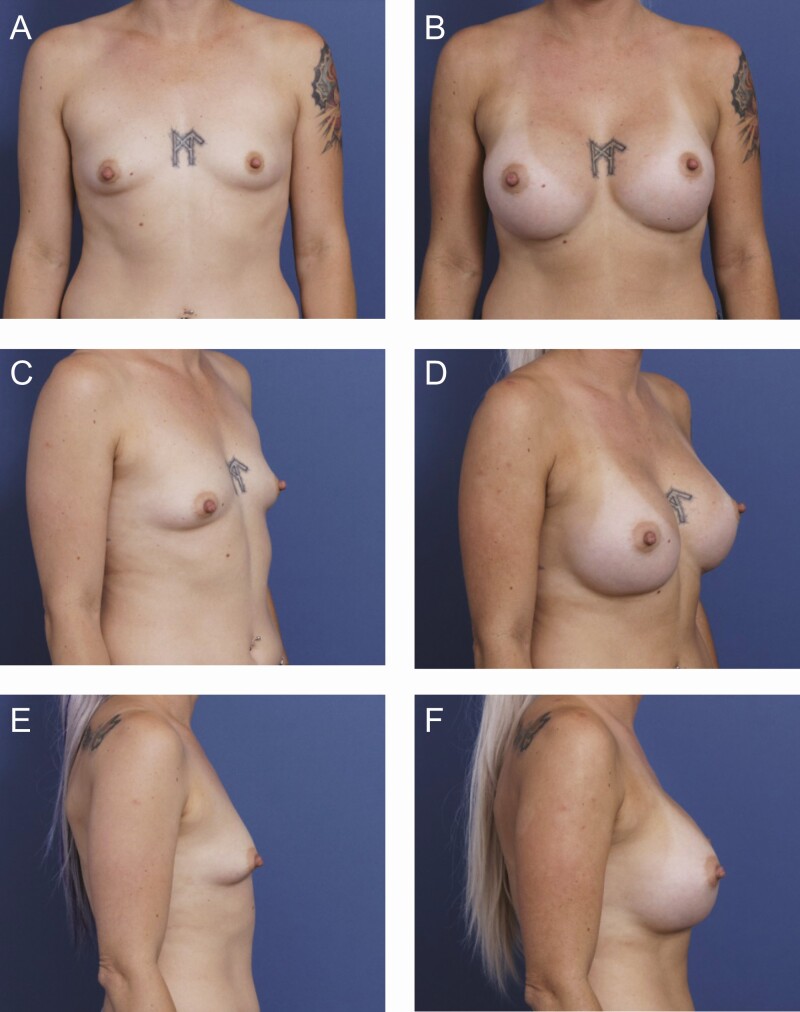
A 36-year-old female patient is shown 9 months after primary breast augmentation using Sientra HSC + High Profile Classic Base-Shaped Implants, 440 mL (Santa Barbara, CA). Shaped implants were selected for this patient based on the constricted lower pole and short nipple-to-fold distance. (A) Frontal, (C) three-quarter, and (E) lateral views are shown preoperatively; and (B) frontal, (D) three-quarter, and (F) lateral views are shown at 9-month follow-up.

**Figure 5.**
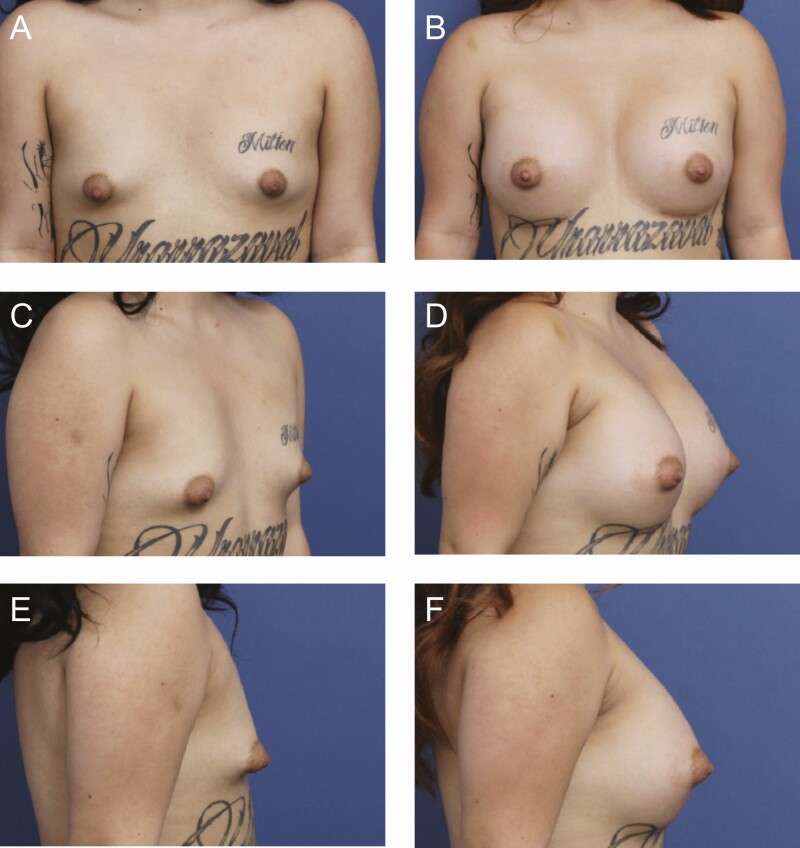
A 24-year-old female patient is shown 6 months after primary breast augmentation using Sientra HSC + High Profile Classic Base-Shaped Implants, 485R and 440L (Santa Barbara, CA). Shaped implants were selected for this patient to correct tuberous shape and construct a lower pole. (A) Frontal, (C) three-quarter, and (E) lateral views are shown preoperatively; and (B) frontal, (D) three-quarter, and (F) lateral views are shown at 6-month follow-up.

One of the unique features of shaped implants is that they are at risk for malrotation. Because of this rare phenomenon, shaped implants have been produced exclusively with textured shells. There is a subset of patients who lack lower pole volume or have tuberous breast deformities that benefit from shaped implants (Figures 4, 5). Even in patients who are agreeable to staged fat grafting to overcome these deficiencies, there are many cases where such patients do not have adequate fat stores. Irrespective of our findings, there are patients with anatomical and physiological limitations that make micro-textured implants more appealing despite their possible associated risks.

With deeper analysis, the smooth implant group trended toward a slightly higher rate of scar malposition in patients who received Allergan’s Natrelle Inspira SoftTouch Smooth Round Gel implants (17.3%) compared with the patients who received Sientra’s HSC + Opus Luxe Smooth implants (12.9%), though this difference was not statistically significant. Of note, patients who were selected to receive Allergan smooth round implants tended to be opting for larger implants (average volume = 458 mL) compared with the patients selected to receive Sientra smooth round implants (average volume = 402 mL); this was a statistically significant difference (*P* value 0.00036) as the senior author tended to use Allergan implants exclusively for larger implant sizes not available by Sientra. Larger breast implants have been found to be more likely to develop malposition or ptosis due to their greater mass placing strain on the pocket over time.^20^ Thus, the authors concluded that this difference was likely not due to a true distinction in the clinical performance of the 2 brands’ smooth implant surfaces, but rather the trend in implant size selected by the patients.

All patients in this study cohort received preoperative counseling during either their consultation or preoperative appointment on both of these potential complications regardless of the implant type they would be receiving. Patients were given a brief synthesis of the existing research on these conditions, the clinical implications of developing either complication (including prevention, treatment, their own unique risk for each depending on their implant surface), and the opportunity to ask the surgeon questions about capsular contracture, BIA-ALCL, and any other potential complications associated with a primary breast augmentation.

Decreased incidence of Baker grades III and IV capsular contracture with textured shells has been observed, though some studies exist to contradict the relationship between texture and decreased capsular contracture^21^. One current theory for this correlation is that the texture of the implant surface causes degradation of the contracted capsule that is beneficial to preventing advanced capsular contracture, while another theory posits that the breast tissue grows into the texture and increases friction over a greater surface area, which reduces synovial-type metaplasia observed in the capsules formed around smooth breast implants and disrupts the planar arrangement of fibroblasts, altering the vectors of contraction.^21-23^ A variety of risk factors for capsular contracture in addition to the use of smooth implants have also been identified, including subglandular placement, the use of a periareolar incision, device size (<355 mL), and surgical bra usage.^1,24^

BIA-ALCL would be a tragic sequela of an elective procedure; however, there are also numerous risks associated with reoperation necessitated by the incidence of major implant malposition or capsular contracture. Regimented follow-up with the patient post-surgery can allow for a quick evaluation and treatment of any sudden expansion or pain that may be associated with a possible complication related to a textured breast implant. Case studies of BIA-ALCL show that it is treatable when detected early, with most cases treated by total capsulectomy yielding a favorable prognosis.^10^ The elimination of the option for board-certified plastic surgeons to utilize micro-textured implants in proper applications would undermine the goal of providing the best outcomes to patients with each individual’s unique history and risk factors properly weighted and taken into account.

While the textured shell may provide a protective effect against capsular contracture, they pose a small risk of BIA-ALCL compared with no risk with the use of smooth implants. Current theories for cancer’s pathogenesis include a possible immune reaction to the silicone of the implant or inflammation caused by the surface texture, with the higher surface area of macro-textured implants providing a larger area available for bacterial growth and subsequent inflammatory response.^9,25,26^ While both capsular contracture and BIA-ALCL are linked with bacterial contamination, Gram-positive bacteria are associated with capsular contracture, whereas Gram-negative bacteria are associated with BIA-ALCL cases.^1,27^ The current hypothesized reason for this difference is that Gram-positive bacteria provide a pathway toward inflammation and fibrosis, leading to capsular contracture, whereas Gram-negative bacteria trigger lymphocyte stimulation.^1,13^ Thus, the current approach to minimizing the risk of BIA-ALCL is reducing Gram-negative bacterial burden through pocket irrigation with antibiotics.

This study cohort was small and took place over less than 2 years and thus did not record any cases of BIA-ALCL or capsular contracture as these conditions typically take years to develop. The main purpose of this study was to evaluate micro-texture’s protective benefit against implant malposition rather than investigate the development of these implant-related complications. Taking into account the current literature defining the risk level of BIA-ALCL with micro-textured implants, the authors believe that their results provide a rationale for the standard use of smooth implants whenever anatomy permits; however, the use of micro-textured implants in more complex augmentations (ie, a case requiring a shaped implant) may present benefits that outweigh the relative risk of BIA-ALCL, such as a reduced risk of reoperation.

**Figure 6.**
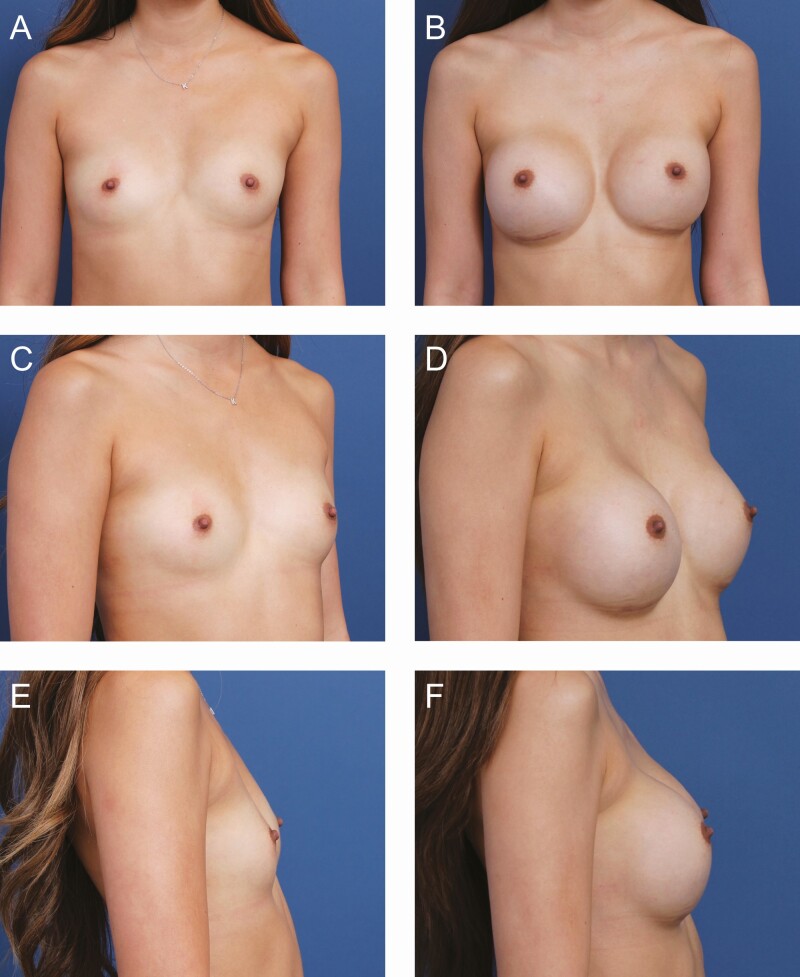
A 21-year-old female patient is shown 7 months after primary breast augmentation using Allergan Natrelle Inspira SoftTouch SSF Smooth Round Implants, 345 mL (Irvine, CA), demonstrating moderate malposition (2 mm L, 2.75 mm R). (A) Frontal, (C) three-quarter, and (E) lateral views are shown preoperatively; and (B) frontal, (D) three-quarter, and (F) lateral views are shown at 7-month follow-up.

**Figure 7.**
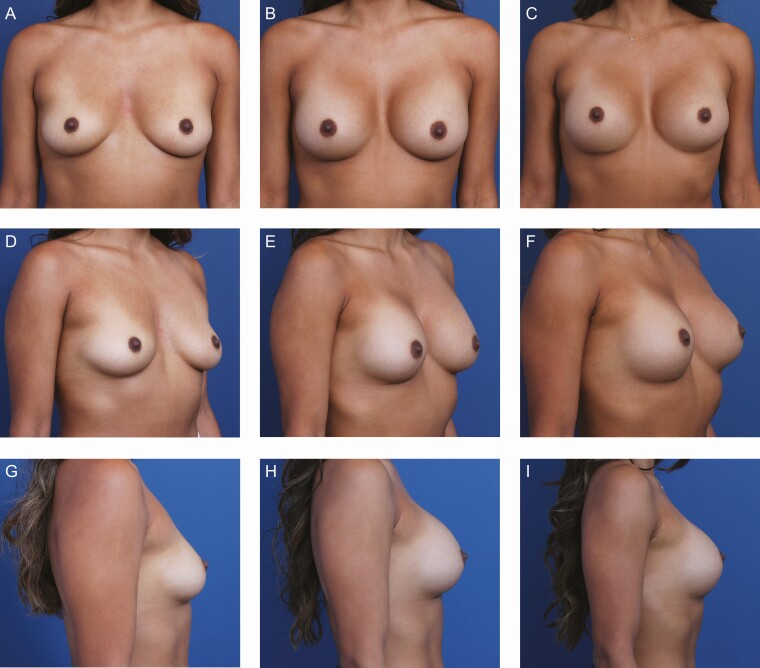
A 28-year-old female patient’s progress is shown after primary breast augmentation using Sientra HSC + High Profile Round Micro-textured Implants, 350 mL (Santa Barbara, CA). The patient demonstrates enduring stability of bilateral pockets and scar position with micro-textured implants. (A) Frontal, (D) three-quarter, and (G) lateral views are shown preoperatively; (B) frontal, (E) three-quarter, and (H) lateral views shown at 6-month follow-up; and (C) frontal, (F) three-quarter, and (I) lateral views are shown at 12-month follow-up.

In patients where a similar aesthetic and functional outcome could be achieved using round smooth silicone gel breast implants, the senior author always opted to use the smooth shell unless a micro-textured breast implant was requested by the patient. Reasons cited by patients for requesting a micro-textured implant included more durable and long-lasting results through better pocket control (Figures 6, 7). Cases in which Sientra’s round micro-textured implants were considered include patients with features of pectus carinatum or pectus excavatum, patients with an inframammary fold that is not discrete or must be moved, or patients with fold instability. In cases where there was an absent or constricted lower pole or overall poor breast shape, Sientra’s shaped micro-textured implants were chosen, especially when the patient had insufficient fat stores for fat grafting to augment soft tissue deficits.

As can be seen, the majority of devices implanted by the senior author were smooth-surfaced implants, because, in a majority of situations, a smooth device can probably yield an equivalent outcome to a textured device with less risk for anaplastic large cell lymphoma; however, the option of a micro-textured implant for select patients was an important tool for meeting individual patient needs. The authors also believe that adherence to a frequent follow-up schedule with all patients, regardless of the implant surface, is crucial in maintaining patient safety so that the development of implant-related complications may be caught early. The patients in this cohort were seen according to the regular follow-up schedule previously described and, after this remedial period, are monitored lifelong with the exception of patients lost to follow-up. Ultimately, the authors believe that this minimizes the risk of using micro-textured implants in qualifying patients. Future studies looking at greater control with internal support matrices may be considered as alternatives to micro-textured implants.^28^

## Conclusions

A board-certified plastic surgeon has greater tools in their armamentarium in the modern era—this comes with a responsibility to understand and maintain the proper usage of these techniques and options. There is a balance to be struck between meeting patient expectations and achieving durable and safe outcomes. We believe that, with proper surgical technique, patient counseling, and implant selection, the micro-textured implants can still be safely used in modern breast augmentation surgery and should be maintained as an option for board-certified plastic surgeons in order to provide individualized results on a patient-by-patient basis.

## References

[1] Calobrace MB, Schwartz MR, Zeidler KR, Pittman TA, Cohen R, Stevens WG. Long-term safety of textured and smooth breast implants. Aesthet Surg J. 2017;38(1):38-48. doi: 10.1093/asj/sjx157 29040370

[2] Baker J . Augmentation mammoplasty. In: Owsley JW Jr, ed. Symposium on Aesthetic Surgery of the Breast: Proceedings of the Symposium of the Educational Foundation of the American Society of Plastic and Reconstructive Surgeons, and the American Society for Aesthetic Plastic Surgery, November 23-26, 1975, Scottsdale, AZ. Mosby; 1978:256-263.

[3] Duteille F, Perrot P, Bacheley MH, Stewart S. Eight-year safety data for round and anatomical silicone gel breast implants. Aesthet Surg J. 2018;38(2):151-161. doi: 10.1093/asj/sjx117 29040345

[4] Randquist C, Gribbe O. Highly cohesive textured form stable gel implants: principles and techniques. In: Hall-Findlay E, Evans G, eds. Aesthetic and Reconstructive Surgery of the Breast. Elsevier; 2010:339-365.

[5] ISO 14607:2018 Annex H: Test for surface characteristics. https://www.iso.org/obp/ui/#iso:std:iso:14607:ed-3:v2:en. Accessed December 10, 2020.

[6] U.S. Food and Drug Administration. The FDA Requests Allergan Voluntarily Recall Natrelle BIOCELL Textured Breast Implants and Tissue Expanders from the Market to Protect Patients: FDA Safety Communication. https://www.fda.gov/medical-devices/safety-communications/fda-requests-allergan-voluntarily-recall-natrelle-biocell-textured-breast-implants-and-tissue. Accessed January 21, 2021.

[7] Deva AK, Adams WP, Jr, Vickery K. The role of bacterial biofilms in device-associated infection. Plast Reconstr Surg. 2013;132(5):1319-1328. doi: 10.1097/PRS.0b013e3182a3c105 23924649

[8] Adams WP, Jr, Conner WC, Barton FE, Jr, Rohrich RJ. Optimizing breast pocket irrigation: an in vitro study and clinical implications. Plast Reconstr Surg. 2000;105(1):334-338; discussion 339; discussion 339. doi: 10.1097/00006534-200001000-00051 10627003

[9] Barr S, Hill EW, Bayat A. Functional biocompatibility testing of silicone breast implants and a novel classification system based on surface roughness. J Mech Behav Biomed Mater. 2017;75:75-81. doi: 10.1016/j.jmbbm.2017.06.030 28697402

[10] Calobrace MB, Stevens WG, Capizzi PJ, Cohen R, Godinez T, Beckstrand M. Risk factor analysis for capsular contracture: a 10-year Sientra study using round, smooth, and textured implants for breast augmentation. Plast Reconstr Surg. 2018;141(4 Suppl):20S-28S. doi: 10.1097/PRS.0000000000004351 29595715

[11] Stevens WG, Calobrace MB, Alizadeh K, Zeidler KR, Harrington JL, d′Incelli RC. Ten-year core study data for Sientra’s Food and Drug Administration-approved round and shaped breast implants with cohesive silicone gel. Plast Reconstr Surg. 2018;141(4 Suppl):7S-19S. doi: 10.1097/PRS.0000000000004350 29595714

[12] Collett DJ, Rakhorst H, Lennox P, Magnusson M, Cooter R, Deva AK. Current risk estimate of breast implant-associated anaplastic large cell lymphoma in textured breast implants. Plast Reconstr Surg. 2019;143(3 Suppl):30S-40S. doi: 10.1097/PRS.0000000000005567 30817554

[13] Loch-Wilkinson A, Beath KJ, Knight RJW, et al Breast implant-associated anaplastic large cell lymphoma in Australia and New Zealand: high-surface-area textured implants are associated with increased risk. Plast Reconstr Surg. 2017;140(4):645-654. doi: 10.1097/PRS.0000000000003654 28481803

[14] Brody GS, Deapen D, Gill P, Epstein A, Martin S, Elatra W. T cell non-Hodgkin’s anaplastic lymphoma associated with one style of breast implants. Presented at: The 89th Annual Conference of the American Society of Plastic Surgeons; October 23-27, 2009; Seattle, WA.

[15] Maxwell GP, Scheflan M, Spear S, Nava MB, Hedén P. Benefits and limitations of macrotextured breast implants and consensus recommendations for optimizing their effectiveness. Aesthet Surg J. 2014;34(6):876-881. doi: 10.1177/1090820X14538635 25024450

[16] Jones P, Mempin M, Hu H, et al The functional influence of breast implant outer shell morphology on bacterial attachment and growth. Plast Reconstr Surg. 2018;142(4):837-849. doi: 10.1097/PRS.0000000000004801 30252806

[17] Hammond D . Long-term clinical performance of Memoryshape® breast implants in breast augmentation and reconstruction: prospective data through 10 years. Presented at: The American Association of Plastic Surgeons Annual Meeting; April 11-14, 2015; Scottsdale, AZ.

[18] Allergan, Inc. Directions for use, Natrelle 410 highly cohesive anatomically shaped silicone-filled breast implants. Allergan; 2015. Updated June 2015. https://media.allergan.com/actavis/actavis/media/allergan-pdf-documents/labeling/natrelleus/410implants/natrelle-410-dfu-l3717rev04.pdf. Accessed January 21, 2021.

[19] Maxwell GP, Van Natta BW, Bengtson BP, Murphy DK. Ten-year results from the Natrelle 410 anatomical form-stable silicone breast implant core study. [published correction appears in Aesthet Surg J. 2015 Nov;35(8):1044]. Aesthet Surg J. 2015;35(2):145-155. doi: 10.1093/asj/sju084 25717116 PMC4399443

[20] U.S. Food and Drug Administration. Risks and complications of breast implants. https://www.fda.gov/medical-devices/breast-implants/risks-and-complications-breast-implants. Accessed January 21, 2021.

[21] Brohim RM, Foresman PA, Hildebrandt PK, Rodeheaver GT. Early tissue reaction to textured breast implant surfaces. Ann Plast Surg. 1992;28(4):354-362. doi: 10.1097/00000637-199204000-00010 1596069

[22] Lista F, Austin RE, Saheb-Al-Zamani M, Ahmad J. Does implant surface texture affect the risk of capsular contracture in subglandular breast augmentation and breast augmentation-mastopexy? Aesthet Surg J. 2020;40(5):499-512. doi: 10.1093/asj/sjz241 31529039

[23] Hall-Findlay EJ . Breast implant complication review: double capsules and late seromas. Plast Reconstr Surg. 2011;127(1):56-66. doi: 10.1097/PRS.0b013e3181fad34d 21200201

[24] Taylor SR, Gibbons DF. Effect of surface texture on the soft tissue response to polymer implants. J Biomed Mater Res. 1983;17(2):205-227. doi: 10.1002/jbm.820170202 6841364

[25] Clemens MW, Medeiros LJ, Butler CE, et al Complete surgical excision is essential for the management of patients with breast implant-associated anaplastic large-cell lymphoma. J Clin Oncol. 2016;34(2): 160-168. doi: 10.1200/JCO.2015.63.3412 26628470 PMC4872006

[26] Roden AC, Macon WR, Keeney GL, Myers JL, Feldman AL, Dogan A. Seroma-associated primary anaplastic large-cell lymphoma adjacent to breast implants: and indolent lymphoproliferative disorder. Mod Pathol. 2008;21(4):455-463. doi: 10.1038/modpathol.3801024 18223553

[27] Rastogi P, Riordan E, Moon D, Deva AK. Theories of etiopathogenesis of breast implant-associated anaplastic large cell lymphoma. Plast Reconstr Surg. 2019;143(3 Suppl):23S-29S. doi: 10.1097/PRS.0000000000005566 30817553

[28] Chiemi JA, Kelishadi SS. Polydioxanone internal support matrix: a rationale for prophylactic internal bra support in breast augmentation. 2022:4:ojac021. doi: 10.1093/asjof/ojac021. PMC9113087 35592182

